# The Gaze Cueing Effect and Its Enhancement by Facial Expressions Are Impacted by Task Demands: Direct Comparison of Target Localization and Discrimination Tasks

**DOI:** 10.3389/fpsyg.2021.618606

**Published:** 2021-03-11

**Authors:** Zelin Chen, Sarah D. McCrackin, Alicia Morgan, Roxane J. Itier

**Affiliations:** Department of Psychology, University of Waterloo, Waterloo, ON, Canada

**Keywords:** gaze cueing, facial expressions, localization task, discrimination task, cognitive resources

## Abstract

The gaze cueing effect is characterized by faster attentional orienting to a gazed-at than a non-gazed-at target. This effect is often enhanced when the gazing face bears an emotional expression, though this finding is modulated by a number of factors. Here, we tested whether the type of task performed might be one such modulating factor. Target localization and target discrimination tasks are the two most commonly used gaze cueing tasks, and they arguably differ in cognitive resources, which could impact how emotional expression and gaze cues are integrated to orient attention. In a within-subjects design, participants performed both target localization and discrimination gaze cueing tasks with neutral, happy, and fearful faces. The gaze cueing effect for neutral faces was greatly reduced in the discrimination task relative to the localization task, and the emotional enhancement of the gaze cueing effect was only present in the localization task and only when this task was performed first. These results suggest that cognitive resources are needed for gaze cueing and for the integration of emotional expressions and gaze cues. We propose that a shift toward local processing may be the mechanism by which the discrimination task interferes with the emotional modulation of gaze cueing. The results support the idea that gaze cueing can be greatly modulated by top-down influences and cognitive resources and thus taps into endogenous attention. Results are discussed within the context of the recently proposed EyeTune model of social attention.

## Introduction

Orienting one’s attention to the direction of another person’s gaze is called “gaze cueing” (Frischen et al., 2007; Bayliss et al., 2013), an ability thought to be critical for successful social interactions. Gaze cueing is believed to facilitate joint attention, the sharing of attention between two individuals toward the same object (Argyle and Cook, 1976; Baron-Cohen et al., 1985). Alterations in gaze cueing are associated with both subclinical (Bayliss et al., 2005; Lassalle and Itier, 2015a; McCrackin and Itier, 2019) and clinical autistic traits (Uono et al., 2009; Gillespie-Lynch et al., 2013) and with social impairment in neurotypical populations (Hayward and Ristic, 2017). Gaze cueing is typically studied using an adaptation of Posner’s cueing task (Posner, 1980), in which a central face either looks to the same side (congruent) or the opposite side (incongruent) as a target. Participants’ target detection is typically faster during congruent than incongruent trials and this reaction time difference, called the gaze cueing effect, is thought to reflect the magnitude of attentional orienting by gaze.

Understanding facial expressions of emotion is another important skill in navigating social interactions, as they give clues about the emotional state of the person expressing them (Ekman and Friesen, 1971). When integrated with gaze cues, emotional expressions can inform an observer about how the gazer is reacting to the surrounding environment. For example, a person looking to the right and expressing fear suggests danger in that direction. The ability to integrate and understand these cues has thus been interpreted as an important survival mechanism (Mathews et al., 2003; Tipples, 2006). The impact of emotional expressions on gaze-cueing has been studied by comparing the magnitude of the gaze cueing effect for emotional faces to that of the gaze cueing effect for neutral faces. Many studies have found larger gaze cueing effects for fearful (Pecchinenda et al., 2008; Bayless et al., 2011; Lassalle and Itier, 2013, 2015a,b; Neath et al., 2013; Dawel et al., 2015; McCrackin and Itier, 2018, 2019) and angry (Holmes et al., 2006; Lassalle and Itier, 2015b) faces relative to neutral or happy ones. These findings have an intuitive appeal, as these are expressions that imply potential threat in the environment, which would arguably be adaptive to quickly attend to. However, enhanced gaze cueing effects have also been reported for expressions that do not necessarily convey threat such as surprised (Bayless et al., 2011; Lassalle and Itier, 2013, 2015b) or disgusted expressions (Pecchinenda et al., 2008). Finally, happy expressions may convey potential reward, which is also adaptive to respond to quickly. Larger gaze cueing for happy faces compared to other expressions has indeed been reported, either restricted to female faces (Hori et al., 2005) or when targets were objects of positive (congruent) valence (Bayliss et al., 2010). Two recent studies also reported enhanced gaze cueing by happy faces compared to neutral faces (McCrackin and Itier, 2018; McCrackin and Itier, 2019) and suggested that, in addition to expressions that convey threat or uncertainty, an enhanced gaze cueing effect can also be induced by positive expressions and their potential reward, though this enhancement is of smaller magnitude than the one found for fear. Many of these studies have found that the enhancement of gaze cueing by facial expressions was driven essentially by shorter reaction times for emotional than neutral faces in the congruent trials, supporting the idea of a truly faster orienting of attention in the direction of perceived gaze when the face expressed an emotion (Bayless et al., 2011; Lassalle and Itier, 2013, 2015a,b; Neath et al., 2013; McCrackin and Itier, 2018, 2019; but see Pecchinenda and Petrucci, 2016, for an effect of anger driven by incongruent trials).

While emotional modulation of gaze cueing has now been reported in many studies, other studies have failed to find a significant enhancement of gaze cueing by emotion (see Frischen et al., 2007; McCrackin and Itier, 2019; Dalmaso et al., 2020 for reviews). The field is becoming increasingly aware of methodological factors that may help to explain these inconsistencies. These include slight modifications to the cueing paradigm such as the dynamic stimulus sequence used (Lassalle and Itier, 2015b) or the cue-target interval (Graham et al., 2010), as well as individual variables such as gender and autistic traits (e.g., Bayliss et al., 2005; Lassalle and Itier, 2015a; Hayward and Ristic, 2017; McCrackin and Itier, 2019). The main goal of the present study was to investigate the effects of task demands, a potentially important moderating factor that has received little attention to date. We first describe evidence suggesting that task demands likely influence the gaze cueing effect and its modulation by emotion, before discussing the other known methodological factors.

There is prior evidence that increasing task demands can reduce the gaze cueing effect, though most studies on this subject have only used neutral faces. These studies had participants complete a gaze cueing task alongside concurrent tasks varying in cognitive demands. Earlier studies found that the neutral gaze cueing effect was not impacted by concurrent cognitive load from tasks including working memory retention of a five-digit sequence (Law et al., 2010), reciting the numbers from one to nine in order (Hayward and Ristic, 2013), building and remembering fill patterns of a 3 × 5 matrix (Law et al., 2010), and perceptual loads imposed by Rapid Serial Visual Presentation in which participants identified a target number either cued by eye-gaze or not (Xu et al., 2011). However, Bobak and Langton (2015) later suggested that these tasks may not be sufficiently demanding to compete with cognitive resources needed for gaze cueing. A random number generation task was suggested as an alternative (Bobak and Langton, 2015), as it places high demands on working memory resources (Vandierendonck et al., 2004; Towse and Cheshire, 2007) and requires active attention throughout the task period. Indeed, it was found that the neutral gaze cueing effect was present when participants listed numbers 1–9 in order, but not when they randomly generated the numbers instead (Bobak and Langton, 2015). This demonstrated that cognitive demand might need to be quite high to produce a reduction in the gaze cueing effect for neutral faces. In contrast, the emotional modulation of gaze cueing may be more easily impacted by task demands, given that it requires the integration of facial expression and gaze cues (Haxby et al., 2000; Fichtenholtz et al., 2009). To the best of our knowledge, only one prior study has investigated the impact of cognitive demands on gaze-cueing with emotional faces. Pecchinenda and Petrucci (2016) found that the gaze cueing effect for happy faces was absent in a high cognitive load condition (counting backward by 7) but present in a low load condition (counting forward by 2). Interestingly, the opposite pattern emerged with angry faces, for which the gaze-cueing effect was higher in the high load condition (though the effect was present in both load conditions). Cueing by neutral faces was absent in both load conditions but present in a no-load condition (Pecchinenda and Petrucci, 2016).

Collectively, the studies by Bobak and Langton (2015) and Pecchinenda and Petrucci (2016) suggest that increasing the load of the cognitive control system may disrupt gaze cueing in response to neutral faces, and may impact the emotional modulation of gaze-cueing, although the direction of this impact remains unclear. Importantly, in these studies, cognitive load was manipulated through the use of tasks administered concurrently with the gaze-cueing task. However, the demands imposed by the actual gaze cueing task itself can vary in terms of difficulty and cognitive resources that may also impact gaze cueing and its modulation by facial expressions of emotion. For example, Pecchinenda et al. (2008) reported an increase in gaze cueing with fearful and disgusted expressions when the task required participants to evaluate the valence of target words (Experiment 1), but not when the task required the upper vs. lower case discrimination of these same words (Experiment 2). Similarly, when the task required participants to semantically categorize target pictures, no effect of emotion was seen on gaze cueing (Bayliss et al., 2007) but when the task required localization of target objects, the gaze cueing was boosted for happy faces, although only when the targets were also of positive valence (Bayliss et al., 2010). While these studies suggest that the type of gaze cueing task used might be critical, none of them directly compared different tasks in the same participants. This is an essential aspect given the individual variability, which is known to impact emotional gaze cueing (e.g., Bayliss et al., 2005; Lassalle and Itier, 2015a; Hayward and Ristic, 2017; Hayward et al., 2018; McCrackin and Itier, 2019). Here, we chose to directly compare the effects of two main tasks used in the gaze-cueing literature. In gaze cueing localization tasks, the most frequently used, participants determine the side on which the target appears. In gaze cueing discrimination tasks, participants determine which of two different targets (typically two letters) has been presented. The discrimination task arguably places higher cognitive demand than the localization task, as it requires participants to identify the target in addition to detecting its location. We hypothesized that the higher cognitive demands of the discrimination task may interfere with the integration of emotion and gaze cues. In contrast, the lesser demands of the localization task may allow for more effective integration of emotion and gaze cues. These differences may help explain why some previous discrimination studies have failed to find emotional modulation of gaze-cueing.

Indeed, in a recent summary of the parameters and main findings of 15 gaze cueing studies (McCrackin and Itier, 2019, Appendix Table), the significant modulations of gaze cueing by facial expressions were all found in localization tasks (e.g., Lassalle and Itier, 2013, 2015b; McCrackin and Itier, 2018). The discrimination task, however, has not been used as frequently and the results are mixed and confounded by other factors such as the face sequence type. For example, using a discrimination task and faces expressing an emotion before averting their gaze, some studies found that fearful faces enhanced gaze cueing but either only in highly anxious individuals (Mathews et al., 2003; Fox et al., 2007) or with a magnitude that was positively correlated with trait fearfulness (Tipples, 2006). However, neither the fearful modulation nor the correlation with anxiety was replicated when the sequence used concurrent emotion and gaze shift presentations (Holmes et al., 2010). Overall, the discrimination task has seemingly produced more null results than the localization task. In the present study, we investigated the effects of task demands on the gaze cueing effect and its enhancement by facial expressions, by directly comparing a localization task and a discrimination task within the same participants and using identical stimuli and targets (a letter T or L) in both tasks.

Other methodological factors have also been shown to impact the emotional modulation of gaze cueing. The stimulus presentation sequence appears to be a strong modulator of the emotional enhancement of gaze cueing (Lassalle and Itier, 2015b; McCrackin and Itier, 2019). Studies using static stimuli (a face image with averted gaze) have typically reported no emotional modulation (Hietanen and Leppänen, 2003, Experiments 1–4; Holmes et al., 2006, Experiment 3). With dynamic stimuli, in which a series of face images are displayed quickly one after another to give the appearance of the face changing its gaze and emotional expression, the sequence of presentation of the averted gaze and expression seems key, with the strongest enhancement of gaze cueing when the expression follows the gaze shift compared to when it changes before or simultaneously with gaze shifts (Lassalle and Itier, 2015b). This latter sequence (gaze shift followed by facial expression) is perceived as a person reacting to an object in their periphery after they have seen it, which arguably has more ecological validity than the other two sequence types and may facilitate mentalizing processes that can enhance gaze cueing (Capozzi and Ristic, 2020). For this reason, the present study included this “gaze-shift-first-expression-second” dynamic sequence.

The gaze cue-target Stimulus-Onset Asynchrony (SOA) is also thought to be crucial for emotional modulation, because gaze direction and facial expressions seem to be processed at different times before they are integrated (Klucharev and Sams, 2004; Fichtenholtz et al., 2009). While some studies found no effect of emotion on gaze cueing at SOAs less than 300 ms (Graham et al., 2010; Galfano et al., 2011), a recent study suggested that the emotional modulation of gaze cueing can occur at an SOA as short as 200 ms when the gaze-shift-first-emotion-second face sequence is used (McCrackin and Itier, 2018). However, while gaze cueing enhancement for fearful faces was seen at 200 ms SOA in that study, the enhancement for happy faces was not clear. A later study, however, showed that happy faces elicited larger gaze cueing than neutral faces at 500 ms SOA (McCrackin and Itier, 2019). The modulation of gaze cueing by happy faces might thus partly depend on SOA although a direct comparison of short and long SOA is needed. Indeed, emotional modulation by happy expressions might not only be of smaller magnitude than that of fearful expressions, but also may be slower as well, requiring a longer SOA. Moreover, it is currently unknown whether similar effects of emotion and SOA would be found in a discrimination task.

In summary, to the best of our knowledge, the present study is the first to directly compare the emotional modulation of gaze cueing between a discrimination task (the higher cognitive demand condition) and a localization task (the lower cognitive demand condition) within the same participants. We hypothesized that the higher demands in the discrimination task might reduce the gaze cueing effect and would interfere with the integration of the emotion and the gaze cues, resulting in weakened or absent emotional modulation of the gaze cueing effect for the discrimination task relative to the localization task. A secondary goal was to test the effect of SOA on the gaze cueing enhancement by happy expressions. We predicted that, at least in the localization task, fearful expressions would result in the largest gaze cueing effect regardless of SOA, but that happy expressions would enhance gaze cueing only at the longer SOA.

## Materials and Methods

### Ethics Statement

The study was approved by the University of Waterloo Research Ethics Board. Upon arrival to the lab, participants gave written informed consent before participating in the study.

### Participants

Eighty students from the University of Waterloo were recruited to participate in the study. Seventy-one participants were compensated with course credit and $5 for their participation while nine participants received $20. A total of 15 participants were eliminated: five for completing less than half of the study (which was particularly detrimental given the blocked design of the tasks), two for data exceeding 2.5 standard deviations of the group’s response time means (see Data Analyses section), five for having less than 80% accuracy, and three due to technical errors during data collection. This resulted in a final sample of 65 participants (*n* = 65, 36 males, 29 females, *mean age* = 19.8, *SD* = ±1.80). Of these 65 participants, 33 (18 males and 15 females) performed the localization task first and 32 (18 males and 14 females) performed the discrimination task first. Based on our latest studies (McCrackin and Itier, 2018, 2019), we had planned on keeping a final sample of 40 participants per group to maximize power. The last participant was tested right before the COVID-19 pandemic lockdown, and further testing has since been impossible. These sample sizes are nevertheless in accordance with previous studies in the field.^1^

All participants reported living in Canada and/or the United States for the past 5 years to ensure English language competency and consistent cultural exposure. To ensure no significant impairment in recognizing facial emotions, participants were preselected based on their self-reported ability to recognize faces and facial expressions on a 10-point Likert-type scale administered at the beginning of the term (from 0 – extremely poor to 10 – extremely good abilities). We invited only participants with scores from 7 to 10 to participate. Participants also reported no history of neurological or mental illness, daily recreational or psychiatric drug use, or a history of loss of consciousness for longer than 5 min.

### Stimuli and Design

Four females and four males facial identities (#02, 03, 06, 09, 20, 22, 24, and 27) were taken from the NimStim database^2^ (Tottenham et al., 2009), each displaying fearful, happy, and neutral expressions. The pupils of each face were moved to the left and right corners of both eyes to produce an appearance of averted gaze. The mouth regions of the neutral faces with averted gaze were edited to simulate tongue protrusion, creating “neutral-tongue” expressions. These neutral-tongue expressions were added to control for the change in mouth variations seen with fearful and happy expressions while keeping the overall expression neutral (see McCrackin and Itier, 2018 for details on this condition and validation of the neutral tongue stimuli). This was important to provide a neutral movement control for the perceived motion of the face inherent to emotional trials in this type of dynamic design. Each image was cropped to an oval shape, so that the faces were shown without hair or ears. The GNU Image Manipulation Program (GIMP, version 2.10.18) was used for photo editing. All images were converted to greyscale, and the mean pixel intensity (*M* = 0.8024, *SD* = 0.3897) and mean Root Mean Square (RMS) contrast (*M* = 0.00065, *SD* = 0.00236) were equalized using the SHINE toolbox (Willenbockel et al., 2010).

**Figure 1:**
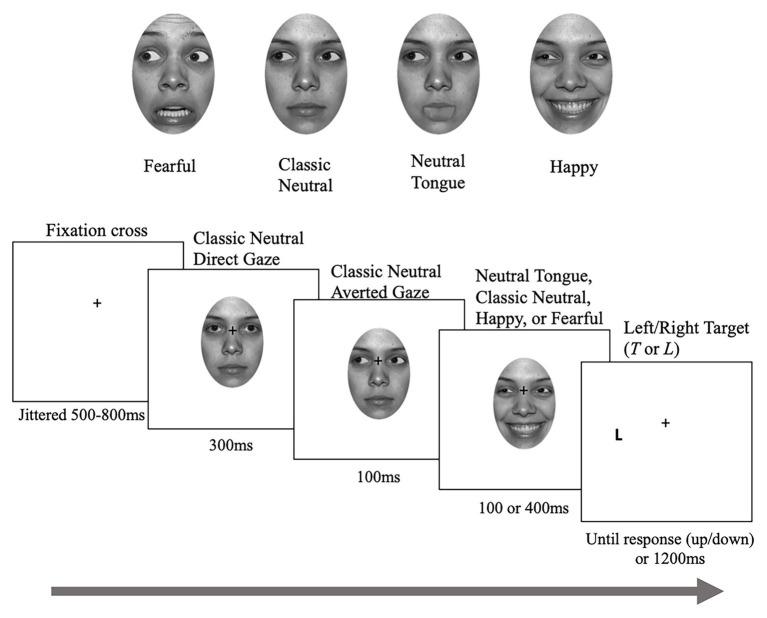
Sample stimuli and trial. Depending on the task, participants either localized or discriminated the targets. Note that the targets were identical for both tasks. The face picture is taken from the NimStim database (Tottenham et al., 2009) for which authorization to publish this particular model’s face has been granted.

In total, there was one direct gaze image of neutral expression and eight averted gaze images [2 gaze directions (left and right) × 4 emotions (classic-neutral, neutral-tongue, happy, and fearful)] for each identity. Images of the same identity were used to create face sequences, each composed of three frames (see Figure 1). First, a neutral face looking straight was presented for 300 ms, followed by a neutral face with an averted gaze to the left or right for 100 ms. The last frame portrayed an averted-gaze that either remained neutral (classic-neutral condition) or displayed a fearful, happy, or neutral-tongue expression, and remained for 100 or 400 ms before the target letter appeared on screen, creating a Stimulus Onset Asynchrony (SOA – time between the gaze shift and the target onset) of 200 or 500 ms. The neutral-tongue condition acted as a second neutral condition in which the apparent motion elicited by the sudden onset of the facial expressions was controlled for.

### Procedure

The experiment was programed using Experiment Builder (SR Research; http://sr-research.com) but eye movements were not recorded. Participants’ head movements were restricted by a chin-rest that ensured a constant distance of 70 cm away from the monitor. The face sequences (vertical visual angle of 12.94° by horizontal visual angle of 8.28°) were presented centrally on a white background while target letters, “T” or “L,” appeared to the left or right of the face 11.303° eccentricity from the center of the screen, centered vertically). Participants were asked to maintain fixation on the fixation cross (0.57° by 0.57°) and to respond as quickly and accurately as possible to the target appearance using the “up” and “down” arrow keys of a keyboard, while not moving their eyes away from the cross. In the *localization* task, participants responded with one key when the target was presented on the left and with the other key when it was presented on the right (regardless of which target it was). In the *discrimination* task, they responded with one key to the “L” and with the other key to the “T.” In both tasks, response key assignments were counterbalanced across participants. Participants were informed that the face gaze direction was not predictive of the target side or target type. Task order was also counterbalanced across participants (33 participants performed the localization task first and 32 did the discrimination task first).

Each trial began with a fixation cross located centrally on the screen and presented randomly for 500, 600, 700, or 800 ms to reduce anticipation effects. Throughout the experiment the fixation cross remained visible and was positioned between the nasion and the nose when a face was presented. The face sequence was then presented, which created the perception of a person looking straight, then shifting their gaze to one side of the screen and either reacting with a facial expression (fearful, happy, and neutral tongue) or not (classic neutral). Immediately after the face sequence ended and disappeared, the uppercase letter “T” or “L” was presented on one side of the screen for a maximum of 1,200 ms or until the participant responded. Participants used the index and middle fingers of their dominant hand to respond. The study took approximately 2 h to complete.

The face gaze was either congruent (looking toward the location where the target would later appear) or incongruent with the target location (in the opposite direction). Left and right gaze trials were averaged within the two gaze-congruency conditions. This within-subject design included a total of 32 conditions: 4 expressions (fearful, happy, classic-neutral, and neutral-tongue) × 2 SOAs (200 and 500 ms) × 2 congruency (congruent and incongruent) × 2 tasks (localization and discrimination). Three blocks were presented in each task, for a total of six blocks and 16 conditions per task. Each block included 512 trials (32 trials per each of the 16 conditions), for a total of 96 trials per condition across the three blocks. Trials were randomly presented with half of the trials congruent and the other half incongruent. There were an equal number of right and left targets, and right and left gaze shifts, in each condition. An additional 16 practice trials were given before the first experimental block of each task.

### Data Analyses

A response was considered incorrect if it was made with the wrong key press, and considered a miss if participants answered after 1,200 ms or gave no response. A response was considered correct if the correct key was pressed and if the response time was less than 2.5 standard deviations away from the mean response time of that condition (computed separately for each task) for each participant (Van Selst and Jolicoeur, 1994). Average correct response times (RTs) and hit rates were calculated for each participant and each experimental condition (for each task separately).

Statistical analyses were conducted using SPSS Statistics 26. The average correct RTs and accuracy rates were analyzed separately using a mixed model analysis of variance (ANOVA) with a between-subject factor of Task Order (2: localization first and discrimination first), and within-subject factors of Task (2: localization and discrimination), Expression (4: fearful, happy, classic-neutral, and neutral-tongue), Congruency (2: congruent and incongruent), and SOA (2: 200 and 500 ms). Due to significant interactions involving Congruency in the RT analysis, we also performed an ANOVA on the gaze cueing scores (RT_incongruent_ − RT_congruent_) using Task Order as a between subject factor and Task, Expression and SOA as within-subject factors. Greenhouse-Geisser corrected degrees of freedom were reported when Mauchly’s Test of sphericity was significant. All follow-up comparisons between expression conditions were carried out with paired *t*-tests. Uncorrected values of *p* are reported for these comparisons for transparency, such that a value of *p* of 0.0083 (0.05/6 for six comparisons) would be considered as significant with the Bonferroni correction. The BayesFactor package in R was used to calculate the Bayes Factors (*B*_10_) for follow up *t*-tests, which were interpreted based on Lee and Wagenmakers (2014) criteria.

## Results

### Hit Rate Results

Overall accuracy was high, with an average of 93.49% correct responses (*SD* = 4.05%) and 6.51% of trials lost due to incorrect button presses, misses, or responses past the time cut-off (1,200 ms).

There was no main effect of Task Order and no interactions with that factor, so analyses were re-run without it included. A main effect of Task [*F*(1, 64) = 27.35; *MSE* = 127.47; *p* < 0.001; *n_p_*^2^ = 0.299) was due to higher hit rates in the localization task (*M* = 94.78%, *SD* = 0.45) than in the discrimination task (*M* = 92.19%, *SD* = 0.66), reflecting that the discrimination task was slightly more difficult. A main effect of Congruency [*F*(1, 64) = 8.18; *MSE* = 8.11; *p* < 0.01; *n_p_*^2^ = 0.113] was also observed, driven by slightly higher accuracy in congruent trials (*M* = 93.66%, *SD* = 0.49) than incongruent trials (*M* = 93.31%, *SD* = 0.52).

A Task by Expression interaction [*F*(3, 192) = 3.28; *MSE* = 5.29; *p* = 0.022; *n_p_*^2^ = 0.049] revealed an effect of Expression in the discrimination task [*F*(3, 192) = 3.37; *MSE* = 6.01; *p* = 0.02; *n_p_*^2^ = 0.05] but not in the localization task [*F*(3, 192) = 0.45; *p* = 0.72; *MSE* = 4.46; *n_p_*^2^ = 0.007]. Follow-up paired comparisons between each expression in the discrimination task revealed only a difference between classic-neutral and neutral-tongue conditions [*t*(64) = 3.26, *p* = 0.002], with slightly lower hit rate for the neutral tongue condition (*M* = 91.88, *SD* = 0.68) than the classic neutral condition (*M* = 92.55, *SD* = 0.66). There were no other effects or interactions.

### Response Time Results

**Figure 2:**
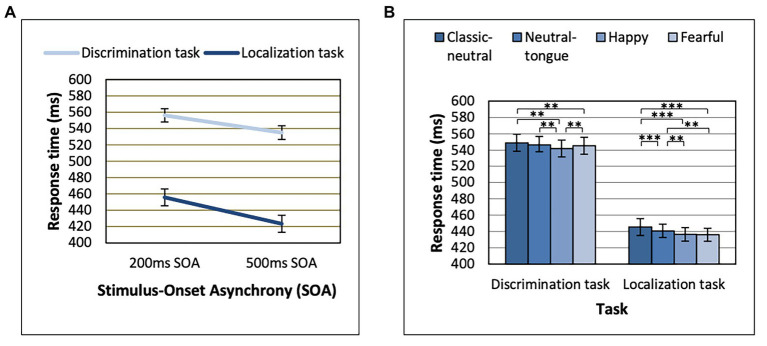
Reaction times displayed for each task as a function of **(A)** Stimulus-Onset Asynchrony (SOA) and **(B)** Expression. ^**^p < 0.01 and ^***^p < 0.001. Error bars represent standard error of the mean.

Task Order effects were only seen in interaction with Congruency effects, which we come back to later (see section “Gaze Cueing Effect Results” on the gaze cueing effect). Response time analyses here were thus re-run without Task Order. Response times were longer in the discrimination task relative to the localization task [main effect of Task, *F*(1, 64) = 160.16; *MSE* = 36391.02; *p* < 0.001; *n_p_*^2^ = 0.714], and in the 200 ms SOA relative to the 500 ms SOA condition [main effect of SOA, *F*(1, 64) = 228.05; *MSE* = 1624.17; *p* < 0.001; *n_p_*^2^ = 0.781]. The effect of SOA was also stronger in the localization task [*F*(1, 64) = 213.62; *MSE* = 1268.89; *p* < 0.001; *n_p_*^2^ = 0.769] than in the discrimination task [*F*(1, 64) = 160.53; *MSE* = 720.34; *p* < 0.001; *n_p_*^2^ = 0.715], as revealed by an SOA by Task interaction [*F*(1, 64) = 44.66; *MSE* = 365.06; *p* < 0.001; *n_p_*^2^ = 0.411; Figure 2A].

A main effect of Expression was found [*F*(3, 192) = 25.39; *MSE* = 253.94; *p* < 0.001; *n_p_*^2^ = 0.284], which was modulated by Task [Expression by Task interaction, *F*(3, 192) = 4.20; *MSE* = 174.75; *p* = 0.007; *n_p_*^2^ = 0.062; Figure 2B]. The Expression effect was stronger in the localization task [*F*(3, 192) = 22.06; *MSE* = 227.87; *p* < 0.001; *n_p_*^2^ = 0.256] than in the discrimination task [*F*(3, 192) = 10.72; *MSE* = 200.82; *p* < 0.001; *n_p_*^2^ = 0.143; Figure 2B]. In the localization task, both fearful and happy expressions (which did not differ, *p* = 0.693) yielded shorter RTs than both classic-neutral and neutral-tongue conditions [fearful-classic-neutral comparison *t*(64) = 6.85, *p* < 0.001, *B*_10_ = 3373274; happy-classic-neutral comparison *t*(64) = 6.49, *p* < 0.001, *B*_10_ = 851128.6; fearful-neutral-tongue comparison *t*(64) = 3.41, *p* = 0.001, *B*_10_ = 23.36; and happy-neutral-tongue comparison *t*(64) = 3.53, *p* < 0.001, *B*_10_ = 32.77]. RTs in the neutral-tongue conditions were also shorter than the classic-neutral conditions [*t*(64) = 3.85, *p* < 0.001, *B*_10_ = 83.92]. In the discrimination task, happy expressions elicited the shortest RTs [happy-classic-neutral comparison *t*(64) = 5.26, *p* < 0.001, *B*_10_ = 33023.04; happy-neutral-tongue comparison *t*(64) = 3.54, *p* < 0.001, *B*_10_ = 33.72; and happy-fearful comparison *t*(64) = 2.79, *p* = 0.007, *B*_10_ = 4.67]. Fearful expressions yielded shorter RTs than classic-neutral expressions [*t*(64) = 2.97, *p* = 0.004, *B*_10_ = 7.27] but did not differ from the neutral-tongue expressions [*t*(64) = 0.90, *p* = 0.374, *B*_10_ = 0.199]. Classic-neutral and neutral-tongue conditions did not differ [*t*(64) = 2.205, *p* = 0.031, *B*_10_ = 1.30]. Table 1 displays the mean RTs for each expression seen in both tasks.

**Table 1:** Mean reaction times (in ms) and gaze-cueing effects (GCE, in ms) for each Expression, SOA, and congruency condition (standard deviations in parentheses).

	**Localization task**				
Order	SOA – Congruency	Classic neutral	Neutral tongue	Happy	Fearful
Presented first	200 ms – Congruent	451.730 (11.788)	443.016 (11.175)	439.682 (11.827)	432.685 (11.202)
	200 ms – Incongruent	468.220 (11.666)	469.395 (11.805)	459.366 (11.976)	461.570 (11.534)
	200 ms – GCE	16.490 (4.023)	26.378 (3.422)	19.684 (3.337)	28.886 (3.280)
	500 ms – Congruent	415.276 (12.682)	411.417 (11.791)	407.782 (12.507)	400.890 (11.682)
	500 ms – Incongruent	435.645 (12.132)	431.422 (12.303)	427.404 (11.587)	432.490 (11.770)
	500 ms – GCE	20.370 (3.372)	20.005 (3.945)	19.622 (4.353)	31.600 (4.731)
Presented second	200 ms – Congruent	456.010 (11.970)	454.258 (11.348)	449.225 (12.010)	450.646 (11.375)
	200 ms – Incongruent	468.868 (11.847)	466.861 (11.988)	460.438 (12.162)	461.101 (11.712)
	200 ms – GCE	12.858 (4.085)	12.603 (3.475)	11.213 (3.389)	10.455 (3.331)
	500 ms – Congruent	429.217 (12.879)	418.273 (11.974)	414.42 (12.701)	414.731 (11.864)
	500 ms – Incongruent	438.195 (12.320)	432.058 (12.494)	433.627 (11.767)	433.772 (11.952)
	500 ms – GCE	8.978 (3.425)	13.785 (4.006)	19.206 (4.420)	19.041 (4.805)
	**Discrimination task**				
Order	SOA and Congruency	Classic neutral	Neutral tongue	Happy	Fearful
Presented second	200 ms – Congruent	551.031 (14.928)	553.700 (14.856)	541.789 (14.457)	549.121 (14.779)
	200 ms – Incongruent	560.562 (14.963)	554.950 (14.263)	552.829 (14.366)	550.588 (14.147)
	200 ms – GCE	9.531 (2.988)	1.250 (3.859)	11.040 (3.328)	1.467 (3.365)
	500 ms – Congruent	525.056 (14.755)	529.229 (14.805)	523.203 (14.454)	525.814 (14.929)
	500 ms – Incongruent	540.430 (15.019)	534.543 (14.547)	532.302 (15.400)	533.672 (15.141)
	500 ms – GCE	15.374 (3.594)	5.314 (3.636)	9.099 (4.528)	7.857 (3.662)
Presented first	200 ms – Congruent	560.942 (15.159)	559.959 (15.086)	555.430 (14.682)	558.561 (15.008)
	200 ms – Incongruent	565.718 (15.195)	564.459 (14.484)	554.035 (14.588)	564.165 (14.367)
	200 ms – GCE	4.775 (3.034)	4.499 (3.919)	−1.395 (3.380)	5.604 (3.417)
	500 ms – Congruent	541.059 (14.983)	533.268 (15.034)	532.564 (14.678)	537.109 (15.160)
	500 ms – Incongruent	546.263 (15.252)	540.341 (14.773)	543.417 (15.639)	542.186 (15.375)
	500 ms – GCE	5.203 (3.650)	7.073 (3.692)	10.853 (4.598)	5.078 (3.719)

There was a typical gaze cueing effect [main effect of Congruency, *F*(1, 63) = 169.41; *MSE* = 464.74; *p* < 0.001; *n_p_*^2^ = 0.729] due to shorter RTs for congruent than incongruent trials. As Congruency interacted with almost every other factor, gaze cueing scores were computed (RT_incongruent_ − RT_congruent_) and were analyzed using a 2 (Task Order) × 2 (Task) × 2 (SOA) × 4 (Expression) mixed model ANOVA. We come back to RTs in the context of the gaze cueing effect when analyzing congruent and incongruent trials separately.

### Gaze Cueing Effect Results

**Figure 3:**
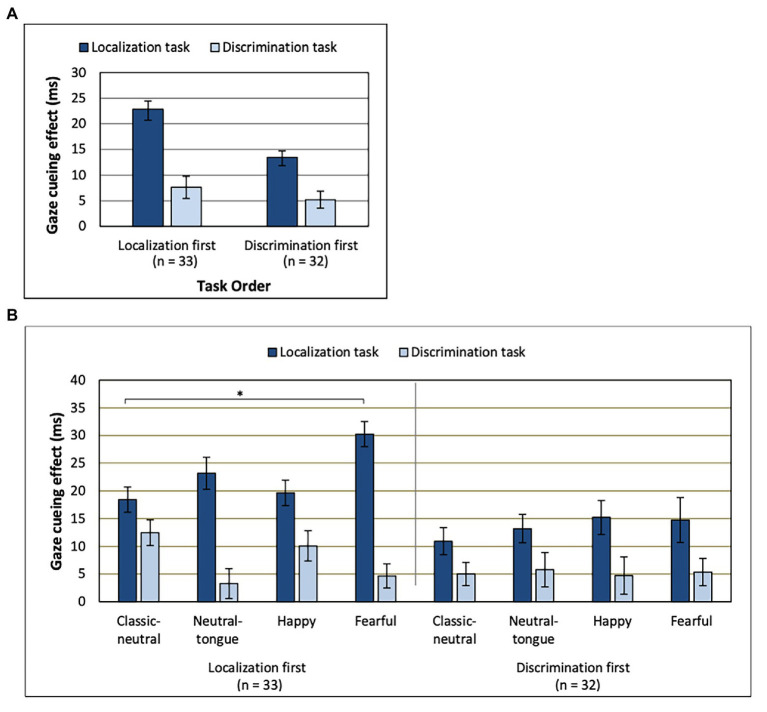
Gaze cueing effect for **(A)** each task depending on which order those were performed in. **(B)** Order, Task, and Expression interaction. Note the lack of clear emotional modulation of gaze cueing in the discrimination task (regardless of task order) and a clear emotional modulation of gaze cueing in the localization task only when that task was presented first. ^*^Uncorrected *p* < 0.0083. Error bars represent standard error of the mean. See Table 1 for mean values.

There was no main effect of SOA [*F*(1, 63) = 3.36; *p* = 0.071; *MSE* = 561.61, *n*^2^*_p_* = 0.051] on the gaze cueing effect. A main effect of task [*F*(1, 63) = 63.49; *MSE* = 568.60; *p* < 0.001; *n_p_*^2^ = 0.502] indicated that there was a stronger gaze cueing effect in the localization task (*M* = 18.20 ms, *SD* = 1.37; Figure 3A) than in the discrimination task (*M* = 6.41 ms, *SD* = 0.997), though the cueing effect was present for both tasks [localization task, congruency effect: *F*(1, 64) = 151.77; *MSE* = 571.85; *p* < 0.001; *n_p_*^2^ = 0.703 and discrimination task, congruency effect: *F*(1, 64) = 41.32; *MSE* = 260.36; *p* < 0.001; *n_p_*^2^ = 0.392].

The Task effect was further modulated by the order of the tasks [Task by Task Order interaction: *F*(1, 63) = 5.53; *MSE* = 568.60; *p* = 0.022; *n_p_*^2^ = 0.081], where the strongest task effect was found in the localization-first order [localization-first order, task effect: *F*(1, 32) = 45.57; *MSE* = 674.84; *p* < 0.001; *n_p_*^2^ = 0.59 and discrimination-first order, task effect: *F*(1, 31) = 19.24; *MSE* = 458.94; *p* < 0.001; *n_p_*^2^ = 0.383]. As seen in Figure 3A, the difference in gaze cueing between the two tasks was larger for the group of participants that performed the localization task first, compared to the group that performed the localization task second.

A Task by Expression interaction [*F*(2.570, 161.904) = 4.18; *MSE* = 442.56; *p* = 0.01; *n_p_*^2^ = 0.062] was qualified by a three-way interaction between Task Order, Task and Expression [*F*(3, 189) = 3.22; *MSE* = 379.12; *p* = 0.024; *n_p_*^2^ = 0.049; Figure 3B]. These results reflected the fact that the emotional modulation of gaze cueing was only seen in the localization task, and only when this task was presented first [effect of Expression for the Localization task in the localization-first group: *F*(3, 96) = 4.28; *MSE* = 434.42; *p* = 0.007; *n_p_*^2^ = 0.118 and effect of Expression for the Localization task in the discrimination-first group: *F*(3, 93) = 0.88; *MSE* = 272.91; *p* = 0.455; *n_p_*^2^ = 0.028]. More precisely, in the localization-first group, fearful expressions elicited the strongest gaze cueing effect, followed by neutral-tongue, happy, and classic-neutral expressions (Table 1). However, only the fearful-classic-neutral comparison was significant using a Bonferroni correction for multiple comparison [fearful-classic-neutral comparison: *t*(32) = 3.03, *p* = 0.005, *B*_10_ = 8.16; fearful-happy comparison: *t*(32) = 2.36, *p* = 0.024, *B*_10_ = 2.055; fearful-neutral-tongue comparison: *t*(32) = 1.99, *p* = 0.055, *B*_10_ = 1.066; happy-classic-neutral comparison: *t*(32) = −0.380, *p* = 0.706, *B*_10_ = 0.120; happy neutral-tongue comparison: *t*(32) = −1.006, *p* = 0.322, *B*_10_ = 0.30; and classic-neutral-neutral-tongue: *t*(32) = −1.632, *p* = 0.113, *B*_10_ = 0.615]. Finally, regardless of task order, there was no impact of Expression on the gaze cueing effect in the discrimination task [*F*(2.582, 162.646) = 1.17; *MSE* = 509.96, *p* = 0.321; *n_p_*^2^ = 0.018; Expression by Task Order: *F*(2.582, 162.646) = 1.67; *MSE* = 509.96, *p* = 0.182; *n_p_*^2^ = 0.026].

The discrimination task elicited RTs that were on average 100 ms longer than those elicited by the localization task. To ensure that this difference in reaction times was not driving the effects obtained, we also computed a gaze-cueing index as a percentage of overall speed {(RT_inc_ − RT_cong_)/[(RT_inc_ + RT_cong_)/2] × 100}, as done previously (Ramon et al., 2010; Dawel et al., 2015; Pecchinenda and Petrucci, 2016). We obtained the same results, with slightly stronger statistics: a main effect of task [*F*(1,63) = 81.67; *MSE* = 29.1; *p* < 0.0001, *n_p_*^2^ = 0.56], a Task by Task Order interaction [*F*(1,63) = 6.24; *MSE* = 29.1; *p* = 0.015; *n_p_*^2^ = 0.090], a Task by Expression interaction [*F*(2.65,167.5) = 5.02; *MSE* = 15.15; *p* = 0.003; *n_p_*^2^ = 0.074], and a three-way interaction between Task Order, Task, and Expression [*F*(3,189) = 3.14; *MSE* = 13.43; *p* = 0.026; *n_p_*^2^ = 0.048]. The only difference was that the effect of SOA now became significant [*F*(1,63) = 5.3; *MSE* = 26.75; *p* = 0.025; *n_p_*^2^ = 0.078], reflecting an overall larger gaze cueing effect for the 500 ms SOA (*M* = 3.11%, *SD* = 0.31) than for the 200 ms SOA (*M* = 2.37%, *SD* = 0.19), but as before, SOA did not interact with any other factor. Follow-up tests for the three-way interaction confirmed that the effect of Expression was significant in the localization task performed first [*F*(3, 96) = 4.86; *MSE* = 19.52; *p* = 0.003; *n_p_*^2^ = 0.132] but was not significant in the localization task performed second [*F*(3, 93) = 1.00; *MSE* = 12.72; *p* = 0.39; *n_p_*^2^ = 0.031] nor in the discrimination task, regardless of Task order [*F*(2.69, 169.96) = 1.05; *MSE* = 14.5, *p* = 0.367; *n_p_*^2^ = 0.016; Expression by Order: *F*(2.69, 169.96) = 1.46; *MSE* = 14.5, *p* = 0.23; *n_p_*^2^ = 0.023]. As before, this effect of Expression in the localization task performed first was driven by largest gaze cueing effect for fearful faces [fearful-classic-neutral comparison: *t*(32) = 3.48, *p* = 0.001, *B*_10_ = 22.97; fearful-happy comparison: *t*(32) = 2.39, *p* = 0.023, *B*_10_ = 2.17; fearful-neutral-tongue comparison: *t*(32) = 2.38, *p* = 0.023, *B*_10_ = 2.13; happy-neutral comparison: *t*(32) = −0.80, *p* = 0.43, *B*_10_ = 0.25; happy-neutral-tongue comparison: *t*(32) = −0.56, *p* = 0.577, *B*_10_ = 0.21; and classic-neutral-neutral-tongue comparison: *t*(32) = −1.52, *p* = 0.137, *B*_10_ = 0.53].

In a series of studies (Bayless et al., 2011; Lassalle and Itier, 2013, 2015a,b; Neath et al., 2013; McCrackin and Itier, 2018, 2019), we have found that the enhancement of gaze cueing by facial expressions was driven most consistently by shorter RTs for emotional than neutral faces in the congruent trials, hereby supporting a true faster orienting of attention in the gaze direction when the face expressed an emotion. In contrast, results for incongruent conditions did not always show a clear picture. However, in another study, the incongruent trials were driving the effect for angry expressions (Pecchinenda and Petrucci, 2016). Thus, in order to better compare the present emotional modulation of gaze-cueing results to previous studies, we also analyzed separately the congruent and incongruent conditions of the localization task (in the localization-first order) using a 4 (expressions) × 2 (SOA) ANOVA.

**Figure 4:**
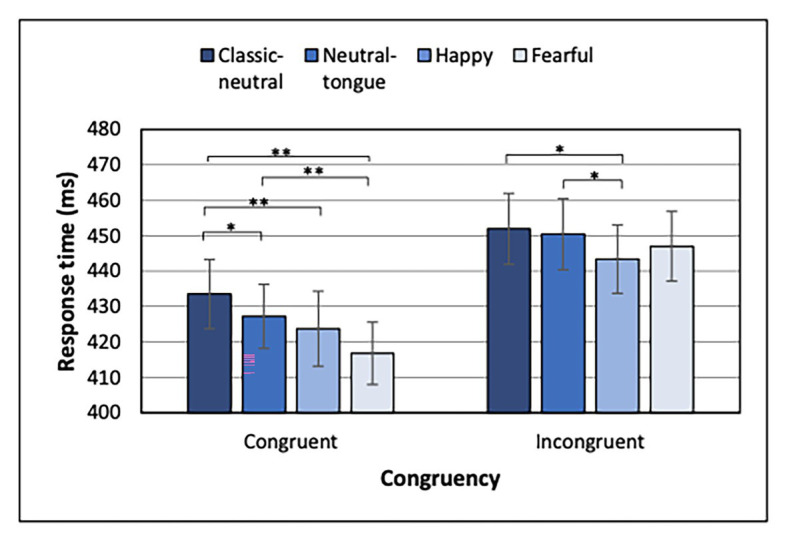
Response times for each expression and congruency condition for the Localization task when Localization task was presented first. ^*^Uncorrected *p* < 0.0083 and ^**^uncorrected *p* < 0.000167. Error bars represent standard error of the mean.

For both congruent and incongruent trials, a typical fore-period effect was present, indicated by shorter RTs in the 500 ms than the 200 ms SOA condition [main effect of SOA for congruent trials: *F*(1, 32) = 50.53; *MSE* = 1417.04; *p* < 0.001; *n_p_*^2^ = 0.612 and incongruent trials: *F*(1, 32) = 66.88, *MSE* = 1068.01; *p* < 0.001; *n_p_*^2^ = 0.676]. Most importantly, a main effect of Expression was found for both congruent [*F*(3, 96) = 15.30; *MSE* = 209.75; *p* < 0.001; *n_p_*^2^ = 0.323] and incongruent [*F*(3, 96) = 4.46; *MSE* = 214.07; *p* = 0.006; *n_p_*^2^ = 0.122] conditions (Figure 4). Uncorrected *p*-values are reported below for paired comparisons, with the same *p* < 0.0083 as significance threshold. For the congruent trials, fearful expressions elicited faster RTs than classic-neutral [*t*(32) = 6.08, *p* < 0.001, *B*_10_ = 20065.59] and neutral-tongue expressions [*t*(32) = 4.69, *p* < 0.001, *B*_10_ = 487.77], but did not differ from happy trials [*t*(32) = 2.37, *p* = 0.024, *B*_10_ = 0.74]. Happy congruent trials elicited faster RTs than classic-neutral congruent trials [*t*(32) = 4.02, *p* < 0.001, *B*_10_ = 86.45], but did not differ from neutral-tongue trials [*t*(32) = 1.41, *p* = 0.169, *B*_10_ = 0.46]. Finally, neutral-tongue congruent trials elicited faster RTs than classic-neutral congruent trials [*t*(32) = 2.82, *p* = 0.008, *B*_10_ = 5.18]. For the incongruent trials, happy expressions elicited faster RTs than classic-neutral [*t*(32) = 3.26, *p* = 0.003, *B*_10_ = 13.72] and neutral-tongue expressions [*t*(32) = 2.95, *p* = 0.006, *B*_10_ = 6.84]. RTs to happy and fearful incongruent trials did not differ [*t*(32) = 1.20, *p* = 0.238, *B*_10_ = 0.36]. Response to fearful, classic-neutral, and neutral-tongue incongruent trials did not differ [fearful-classic-neutral comparison *t*(32) = 2.05, *p* = 0.049, *B*_10_ = 1.18; fearful-neutral-tongue comparison *t*(32) = 1.31, *p* = 0.198, *B*_10_ = 0.41; and classic-neutral-neutral-tongue comparison *t*(32) = 0.69, *p* = 0.493, *B*_10_ = 0.23]. There was no SOA by Expression interaction for either congruency condition.

## Discussion

The ability to orient one’s attention to the direction of another person’s gaze is important for social interactions, and previous work has shown that this orienting can be modulated by the face emotional expression (e.g., Neath et al., 2013; McCrackin and Itier, 2018). However, in addition to individual differences (e.g., Bayliss et al., 2005; Lassalle and Itier, 2015a; Hayward and Ristic, 2017; Hayward et al., 2018; McCrackin and Itier, 2019), methodological factors within the gaze cueing paradigm seem to impact this emotional modulation, such as the face sequence, when using dynamic stimuli (Lassalle and Itier, 2015b) and the Stimulus Onset Asynchrony (SOA; e.g., Graham et al., 2010). The primary goal of the present study was to investigate whether the type of task performed is another such factor that impacts the emotional modulation of the gaze cueing effect. Using the same dynamic face sequence (a gaze shift followed by the expression of emotion) and the same two SOAs, we compared response times and gaze cueing effects between a localization task and a discrimination task, both commonly used in the literature. Importantly, this was done within the same participants, precluding sample variability to account for the differences found. We tested two different groups of participants in which the order of the two tasks was flipped, to account for possible task order effects.

In the localization task, participants detected the location of the target letter (T or L). In the discrimination task, participants needed to identify the target letter in addition to detecting its location. This extra step means that the discrimination task arguably placed higher cognitive demands on participants, and indeed, our results suggest that the discrimination task was more difficult than the localization task, reflected by its longer response times (100 ms longer on average) and lower accuracy rates. Most importantly, across all conditions, the gaze cueing effect was three times as large in the localization task compared to the discrimination task (Figure 3A), reflecting the strong decrease in attention orienting when target discrimination was required. This task difference was not driven by longer reaction times diluting the gaze cueing effect in the discrimination task, because we found the same results using a gaze cueing index calculated as a percentage of reaction times, as done previously (Ramon et al., 2010; Dawel et al., 2015; Pecchinenda and Petrucci, 2016).

This much reduced gaze cueing effect for the discrimination task provides support for the notion that cognitive resources are required for gaze cueing (Bobak and Langton, 2015; Pecchinenda and Petrucci, 2016). What “cognitive resources” means, however, seems to vary with the study design. When cognitive resources are taxed by a secondary task administered concurrently with the gaze cueing task, the impact on gaze cueing may depend on cognitive load and task difficulty. Indeed, Previous studies have found that the gaze cueing effect for neutral faces can be disrupted by a concurrent task that involved continuous manipulation of information in verbal working memory, such as counting forward by 2 or backward by 7 (Pecchinenda and Petrucci, 2016), and generating numbers in random order (Bobak and Langton, 2015). In contrast, tasks that also involved the maintenance of verbal or visuospatial information in working memory, but were less demanding, did not disrupt gaze cueing (Law et al., 2010; Hayward and Ristic, 2013; Bobak and Langton, 2015). Thus, when a secondary task is used, high cognitive load may be required to disrupt gaze cueing for neutral faces.

The picture is less clear when the demands of the gaze cueing task itself are manipulated. In the present study, we assume that the extra cognitive resources of the discrimination task tap into the same resources as those necessary for the localization task, thus differing in quantity and not quality. Gaze cueing requires shifts in spatial attention, so it is reasonable to think that additional demands on visuospatial resources needed to perceptually discriminate the target would reduce the gaze cueing effect. In contrast, Pecchinenda et al. (2008) reported a similar gaze cueing effect for neutral faces whether the task required semantic evaluation of target words (respond to word valence, Experiment 1) or a perceptual discrimination of those words (upper vs. lower case letters, Experiment 2), despite the semantic evaluation task requiring higher cognitive load than the perceptual task, as demonstrated by RTs on average 200 ms longer. This semantic evaluation of the target likely increased the load by taxing different resources than those needed for the perceptual discrimination task, thus increasing reaction times without impacting neutral gaze cueing. Similarly, dual-task load, as in studies using a secondary task, often taps into executive functions and verbal working memory, which might involve different cognitive resources than those required by the gaze cueing task. Here, we showed that the gaze discrimination task strongly reduced the gaze cueing effect for neutral faces (though did not abolish it). Taking the results from these studies together, we propose that the gaze cueing effect to neutral faces can be impacted by both verbal and visuospatial working memory tasks that require continuous attention, but presumably in different ways depending on the exact cognitive resources tapped into. It is yet unclear whether visuospatial demands from a gaze discrimination task can reduce the gaze cueing effect to the same extent as verbal memory and executive function load, an issue that future studies could examine. In any case, the fact that gaze cueing for neutral faces can be considerably diminished by the increase in cognitive resources needed to perform the task supports the view that gaze cueing is not a strictly automatic and exogenous phenomenon but taps into endogenous attention (Jonides, 1981; Frischen et al., 2007; Brignani et al., 2009; Bobak and Langton, 2015; Pecchinenda and Petrucci, 2016; Dalmaso et al., 2020).

We hypothesized that the discrimination task may reduce the emotional modulation of gaze cueing because its higher cognitive demands may interfere with the proper integration of gaze and emotion cues. We found support for this prediction: while gaze cueing by fearful faces was enhanced compared to neutral faces in the localization task, this effect was eliminated in the discrimination task. This finding offers a potential explanation for why previous studies using a letter discrimination task (e.g., Graham et al., 2010, Experiment 3; Holmes et al., 2010) commonly did not find emotional modulation of gaze cueing, and when they did, other factors might have driven these effects, such as the use of a different face sequence combined with participants high in anxiety (e.g., Mathews et al., 2003; Holmes et al., 2006; Fox et al., 2007). We should first note that it is possible that participants cannot maintain proper fixation during discrimination tasks, which to our knowledge has never been tested. If so, participants might move their eyes toward the target and miss the facial expression, preventing the perception of emotion from modulating the gaze cueing effect. However, it has been shown that participants are quite good at maintaining fixation during the localization task (McCrackin et al., 2019) and at debriefing participants did not report having trouble maintaining fixation in either task, which was expected given the target was presented within central vision (11° of eccentricity). It is also unclear how any lack of fixation in the discrimination task could account for the general decrease in gaze cueing, even for neutral faces, unless participants simply missed the cue altogether, in which case, we would expect a complete absence of gaze cueing. Therefore, until future studies can demonstrate that fixation cannot be maintained in the discrimination task, we will concentrate on the other, more interesting idea that the nature of the cognitive resources impacted by the demands of the task might be what impacts emotional modulation.

Our lack of emotional modulation of gaze cueing in the discrimination task is similar to the lack of emotional modulation of gaze cueing reported by Pecchinenda et al. (2008) when the task required the perceptual discrimination of the letter case (upper/lower case) of target words. In both cases, the task was perceptual in nature and elicited comparable reaction times. In contrast, when the task required the discrimination of the same words according to their valence, which was cognitively more demanding than the perceptual task, larger gaze cueing was found for fearful and disgusted expressions compared to neutral and happy expressions (Pecchinenda et al., 2008). In other words, emotional modulation was found under a *high* load condition. Similarly, when using a secondary task concurrently with the gaze cueing task, Pecchinenda and Petrucci (2016) found no emotional modulation of gaze cueing under low load but reported a large increase in gaze cueing for angry faces under high load. They interpreted their findings as reflecting the fact that cognitive control mechanisms (executive functions) would normally suppress interference from emotional faces such that, when those resources are taxed or allocated toward a secondary task, the emotion of the face would now impact the gaze cueing task. Although interesting, this interpretation clearly cannot account for our results, which are in the opposite direction (emotional effects seen in the less demanding task), nor for the fact that only angry faces, but not happy faces, modulated gaze cueing in their study. Interestingly, they also reported that the enhancement of gaze cueing for angry expressions was driven by longer RTs for angry than other expressions in incongruent trials. In contrast, our analyses showed that in the localization task, reaction times were faster for emotional congruent trials compared to neutral congruent trials (which Bayesian factors showed was an extremely strong effect for fearful faces and a strong effect for happy faces). This result has already been reported by many previous studies that also used a localization task without a concurrent secondary task (Bayless et al., 2011; Lassalle and Itier, 2013, 2015a,b; Neath et al., 2013; McCrackin and Itier, 2018, 2019). Clearly, different mechanisms are at play in our discrimination task and in the dual-task used by Pecchinenda and Petrucci (2016) and cognitive load alone is unlikely the reason for our lack of emotional gaze cueing modulation in this task. However, what type of cognitive processes is at play seems to depend on the nature of the task used. What we propose is that the *nature* of the cognitive resources used might be the critical factor. Thus, instead of involving executive functions, we propose that the discrimination task demands themselves shift the way gaze and emotional cues are integrated to allow for the perceptual discrimination of the target. As discussed earlier, the discrimination task requires localization of the target and in addition, its actual perceptual discrimination which presumably taxes the same visuospatial cognitive resources. It is possible that the emotional modulation of gaze cueing was disrupted, because the emotional expression was not processed or integrated as effectively with gaze cues due to this extra demand.

Indeed, there is evidence to suggest that maintaining global precedence, which is standard for interpreting real-world stimuli, requires cognitive resources and that when these resources are not available, people shift toward local processing (Hoar and Linnell, 2013). Emotional expression processing involves a global processing strategy (e.g., Calder et al., 2000; White, 2000) in that it requires the integration of several facial features including not only the size and shape of the eyebrows and eyes but also of the mouth. Local processing induced by diminished cognitive resources may reduce the processing of the emotional expression in the discrimination task, and thus the emotional modulation of gaze-cueing. Indeed, Bayless et al. (2011) showed that, although the neutral gaze-cueing effect can be elicited by eye-regions presented in isolation (but see Burra et al., 2017), the enhancement of this effect by emotional expressions requires the full face, and thus, global processing. Perceptual characteristics, such as sclera size, contribute to the appraisal of the emotion (large and widen eyes are the characteristic of fearful faces), but do not drive the emotional modulation of gaze cueing (Bayless et al., 2011). Furthermore, not only might the increased difficulty of the discrimination task bias toward local processing, but also the identification of target letters in the discrimination task is arguably achieved by feature-based processing, which may also promote switching to a local processing mode. In contrast, emotional modulation may be preserved in the localization task, because low cognitive demands allow the global processing of the emotional expressions and because, as discussed later, integrating the emotional and gaze cues might be the default mode of processing when localizing objects in the environment.

The idea that increased cognitive resources may introduce a bias toward using a more local processing strategy is particularly interesting given the association between autism and both increased cognitive load/use of cognitive resources and detail-focused processing (Happé and Frith, 2006). The Intense World Theory of autism suggests that autistic individuals experience a sensory overflow that acts as a cognitive load and compromises their ability to suppress irrelevant information (Markram and Markram, 2010). The Weak Coherence Theory further suggests that weakness in integration of local information to form global meaning contributes to impairment in social skills (Frith and Happé, 1994; Happé and Frith, 2006; Russell-Smith et al., 2012). Having higher levels of autistic-like traits might thus be associated with higher baseline cognitive load which in turn would compromise the ability to process information globally such as the emotional information derived from facial expressions. This bias toward a local processing strategy has been suggested (Lassalle and Itier, 2015a; McCrackin and Itier, 2019) as a potential mechanism explaining the negative relationship between autistic traits and emotional modulation of gaze-cueing in both neurotypical (Hayward and Ristic, 2017) and clinical populations (Uono et al., 2009; Gillespie-Lynch et al., 2013). In support of this theory, McCrackin and Itier (2019) found a negative relationship between emotional modulation and autistic traits that was driven by the Attention to Detail subscale of the Autism Spectrum Quotient (AQ; Baron-Cohen et al., 2001), which contains questions that tap into a local vs. global processing strategy. That is, the higher the prevalence of autistic traits, and in particular the higher the tendency to focus on details, the smaller the emotional modulation of gaze cueing in a localization task (Lassalle and Itier, 2015a; McCrackin and Itier, 2019). The present research suggests that in neurotypical individuals, a similar decrease in the emotional modulation of gaze cueing can be induced by making minor adjustments to task demands that presumably require a local processing strategy. Further research is needed to confirm that a local processing bias during the discrimination task is really what is driving the cancellation of the emotional modulation of gaze cueing.

Overall, our results make sense in the context of the recently proposed EyeTune model (Dalmaso et al., 2020), which attempts to integrate the vast and complex literature on gaze cueing and views the influence of social variables as central for explaining the controversial findings reported in that literature. According to this model, three main dimensions contribute to the modulation of gaze cueing. The first one is the “situational gain,” which relates to whether the gaze cue leads to personal benefit for the observer. The second dimension is the “individual constraints,” where the observer’s characteristics (e.g., gender and personality traits) modulate gaze cueing. The third dimension includes the contextual factors of the environment such as the use of affectively valenced targets or priming conditions. Given the same participants performed the localization and the discrimination tasks, the individual constraint dimension cannot account for our finding that the discrimination task decreases gaze cueing and abolishes its emotional modulation. However, the other two dimensions seem relevant.

First, the situational gain perspective (the first model dimension) fits with our emotional effects in the localization task. Indeed, in general, integrating the emotion of the face is advantageous for the observer to orient toward the source of an unknown object in the environment, especially when it might be potentially dangerous (e.g., a fearful face looking left suggests a possible danger on the left of the observer). This idea has been put forward to explain the previous emotional modulations of gaze cueing, all reported in localization tasks, where the target was meaningless and non-valenced (Bayless et al., 2011; Lassalle and Itier, 2013, 2015a,b; Neath et al., 2013; McCrackin and Itier, 2018, 2019). This idea is supported by the emotional effects seen on the congruent trials, which argues in favor of a truly faster orienting toward the location signaled by gaze when the face expresses an emotion. This natural tendency to orient toward an unknown source seems to manifest even in lab settings when the participant knows perfectly well that there is no danger, and thus might reflect a default mode of orienting toward the source of the other’s emotion. Note that this spontaneous covert orienting is modulated by endogenous participant characteristics like gender and social skills (e.g., Bayliss et al., 2005; Lassalle and Itier, 2015a; Hayward and Ristic, 2017; Hayward et al., 2018; McCrackin and Itier, 2019) and thus can be viewed as an interaction between the situational gain and the individual constraints.

Second, the contextual factors (the third dimension of the model) may explain the intriguing finding that we obtained regarding the task order effects. Indeed, we found that the difference in gaze cueing effect between the two tasks was largest when the localization task was performed first. Moreover, the emotional modulation of gaze cueing seen in the localization task completely vanished when the localization task was performed after the discrimination task. Although different participants performed the two orders, and thus some individual variability might be at play, the task order effect can be seen as a contextual experimental factor. Exactly what mechanism accounts for this order effect is unclear, and we acknowledge that we did not expect such factor to play in. After using local processing in the discrimination task when performed first, it might simply be either harder to switch back to a global processing in the following localization task or the advantage conveyed by the emotional expression might be reduced (or both). However, for now, the order effect can be conceived as top-down modulations driven by the task demands of the discrimination task when performed first (some sort of carry-over effect).

Finally, we turn to the secondary goal of the present study, which was to investigate the effects of happy expressions on gaze cueing as a function of SOA. Although most studies failed to find a gaze cueing enhancement for happy relative to neutral faces (e.g., Neath et al., 2013; Lassalle and Itier, 2015b), a recent study (using a localization task) reported a significant gaze cueing enhancement for happy compared to neutral expressions with tongue protrusion (“neutral-tongue” expressions) at SOAs ranging between 200 and 700 ms (McCrackin and Itier, 2018, Experiments 2–3). However, the comparison was no longer significant when classic-neutral expressions were included in the design over the range of 200–350 ms SOAs (McCrackin and Itier, 2018, Experiment 4). A later study also demonstrated a gaze cueing effect enhancement for happy relative to classic-neutral faces at an SOA of 500 ms (McCrackin and Itier, 2019). Therefore, a direct comparison between happy and the two neutral expressions at both short and long SOAs was necessary to try to disentangle these effects.

When the localization task was performed first, and in line with the general findings in the literature, fearful faces elicited a greater gaze cueing effect than classic-neutral faces (a strong effect according to Bayes Factors) and neutral-tongue faces (a weak effect; Figure 3B). The cueing effects for neutral-tongue and classic-neutral faces also did not differ, suggesting that the neutral-tongue faces were perceived as neutral and that apparent motion, which was present in the neutral-tongue condition, was unlikely driving the enhanced gaze cueing effect by fearful faces. This idea was supported by the faster response times seen for fearful than both classic-neutral and neutral-tongue faces in congruent trials (both extremely strong effects according to Bayes factors), while no expression difference was found for incongruent trials (Figure 4). Therefore, the expression itself was driving this faster orienting of attention toward the location looked at by fearful faces, rather than the perceived motion elicited by the change in feature position between the neutral and the expression frames in this dynamic sequence. Let us highlight here that although neutral-tongue faces controlled only for apparent motion at the level of the mouth, it has been shown that the mouth area is in fact more diagnostic than the eyes in discriminating facial expressions, including fearful ones (Blais et al., 2012), and that motion of the eyes *per se* does not drive the emotional increase in gaze cueing with fearful faces given the effect is abolished when the eyes move inward so as to be crossed (Bayless et al., 2011).

In contrast to the effect of fearful expressions, the picture was less clear for happy expressions. The cueing effect for happy faces did not differ from either classic-neutral or neutral-tongue conditions (and Bayes factors suggested moderate evidence for a lack of effect), so we were not able to replicate the small gaze cueing enhancement for happy expressions (which was also not significantly different from that to fearful faces when using a Bonferroni correction). The separate analysis of congruent and incongruent trials also depicted a mixed picture. Congruent trials analysis revealed faster RTs for happy compared to classic-neutral expressions but not compared to neutral-tongue (or fearful) faces. That is, happy faces elicited intermediate response times, which could be interpreted as being driven by the perceived motion rather than by the expression itself. This pattern of response deviates from the results of McCrackin and Itier (2018) in which both happy and fearful congruent conditions elicited faster responses than both neutral conditions, a finding that was interpreted as reflecting the true effect of emotion rather than apparent motion, for both happy and fearful faces. The detection of target in incongruent trials, however, requires disengagement of attention from the cued location, and participants displayed faster disengagement from targets cued by happy faces than from targets cued by neutral-tongue or classic-neutral faces. This is, again, only partially replicating McCrackin and Itier (2018) results, in which disengagement from happy faces was also faster than disengagement from fearful faces. Nonetheless, results by both McCrackin and Itier (2018) and the present study suggest that shorter RTs to happy incongruent trials may explain the lack of gaze cueing increase for happy compared to neutral trials (moderate to strong effects according to the Bayes factors).

The medium sample size of the current study likely contributes to the lack of gaze cueing increase with happy expressions relative to classic-neutral faces. Due to its small effect size, a large sample may be needed to uncover this increase (McCrackin and Itier, 2018, 2019). In fact, the happy-classic-neutral difference at 500 ms SOA was only found with a large sample of 148 participants (McCrackin and Itier, 2019). In contrast, only 33 participants performed the localization task first and showed emotional modulation of gaze cueing in the present study. The small effect size of gaze cueing enhancement by happy faces, combined with the medium sample size of the present study, might have prevented us from detecting an enhanced gaze cueing effect by happy expressions, an idea supported by the power analysis that we performed *post-hoc* (using the Cohen’s *d* values obtained from the large sample in McCrackin and Itier, 2019), which suggested that we were indeed underpowered for the happy expressions (power = 0.0668). In any case, the present study did not find any support for the idea that the enhancement of gaze cueing by happy faces might be seen only at longer SOA. Future studies should include a larger sample size to elucidate further the potential enhancement of gaze cueing by happy faces.

In conclusion, the present study provided further evidence that, when using a localization task (alone or before another task), fearful faces enhance gaze cueing at both short (200 ms) and long (500 ms) SOAs and that this enhancement is driven by the emotional content of the face, rather than by apparent motion. For this emotional expression, and using this particular gaze-shift-first-emotion-second face sequence, SOA does not matter. Most importantly, the results demonstrate that the use of a target discrimination task can dramatically reduce the gaze cueing effect and completely abolish the emotional modulation of gaze cueing. We propose that the visuospatial cognitive demands of this task interfere with the integration of emotional and gaze cues and may promote bias toward a local processing strategy, resulting in a lack of emotional modulation of gaze cueing (assuming that participants could fixate properly). Further empirical evidence is needed to support this argument. Overall, our results, including the task order effects, can be accounted for by the various social dimensions of the recently proposed EyeTune model of social attention (Dalmaso et al., 2020). Clearly, gaze cueing and its emotional modulation can be modulated by top-down processes, supporting the view that they tap into endogenous attention processes.

## Data Availability Statement

The datasets presented in this study can be found in online repositories. The names of the repository/repositories and accession number(s) can be found at: https://osf.io/6sd4f/?view_only=70bdebcbc248462486ec2fe4eaa2ed07.

## Ethics Statement

The studies involving human participants were reviewed and approved by the Research Ethics Board at the University of Waterloo. The patients/participants provided their written informed consent to participate in this study. Written informed consent was obtained from the individual(s) for the publication of any potentially identifiable images or data included in this article.

## Author Contributions

SM, AM, and RI designed the experiment. SM and AM programmed it. ZC and AM recruited and tested participants and analyzed the data under guidance from RI and SM. ZC, SM, AM, and RI interpreted the data and wrote the manuscript. All authors contributed to the article and approved the submitted version.

### Conflict of Interest

The authors declare that the research was conducted in the absence of any commercial or financial relationships that could be construed as a potential conflict of interest.

## Footnotes

^1^Power analyses were computed *post-hoc* in G*Power 3.1.9.7 using the Cohen’s *d* effect size estimates for the happy (*d* = 0.43) and fearful (*d* = 0.74) emotional enhancements of gaze cueing from McCrackin and Itier (2019). We used the sample size from our localization first group (*n* = 33), as this was the only group that showed emotional modulation. These analyses indicated that our power to detect a difference between the gaze cueing effect for fearful and neutral faces was 0.985, while our power to detect a difference between the gaze cueing effect for happy and neutral faces was 0.668.

^2^Development of the MacBrain Face Stimulus Set was overseen by Nim Tottenham and supported by the John D. and Catherine T. MacArthur Foundation Research Network on Early Experience and Brain Development. Please contact Nim Tottenham at tott0006@tc.umn.edu for more information concerning the stimulus set.
